# An Update on Known Rare Rhnull Phenotype Individuals in Iran

**Published:** 2019-01-01

**Authors:** Ehsan Shahverdi, Marcel Baschin, Mostafa Moghaddam

**Affiliations:** 1 Department of Transfusion Medicine, Institute of Immunology and Transfusion Medicine, University Medicine Greifswald, Greifswald, Germany; 2 Blood Transfusion Research Center, High Institute for Research and Education in Transfusion in Medicine, Tehran, Iran

**To the Editor**

We recently published an article on a rare Rhnull phenotype in a sibling which was detected as a part of a difficult sample work-up for red cell antibody screening and identification. It was the first report of two siblings with Rhnull syndrome living in Iran entitled " First report of known rare Rh null phenotype individuals in Iran" in your prestigious journal^   1 ^.

Iran is a member of the International Society for Blood Transfusion (ISBT) working party on rare donors since 2010^     2 ^. Recognition of individuals with Rhnull phenotype was reported to the working party in order to be added to the list of countries with Rhnull donors. 

Iranian database of rare blood groups is followed by international researchers and is important for the Rare Donor Working Party of the International Society of Blood Transfusion (ISBT). Iran is a country with a relatively high prevalence of rare phenotypes and further new blood groups were newly identified. 

In our published paper, we reported a case study of siblings (a 43-year-old female and her brother) who were strongly suspicious of presenting a rare Rhnull phenotype. Their extended RBC phenotyping showed that they were negative for D, C, E, c, e antigens. We now found out that the brother passed away of unknown causes a few months ago. This means that now we have only one person with this very rare blood group, which raised our concern for supplying compatible blood at the time of need, since the deceased brother was the only donor for his sister. There are two units of frozen RBC donated previously by him in stock. We are almost sure that blood transfusion services might face challenges to provide compatible blood to this individual who lacks all Rh antigens.

In conclusion, at present, there is only one individual (a female) patient with the rare Rhnull phenotype in Iran. 
